# Device‐Based Physical Activity and Low‐Grade Inflammation in People With Multimorbidity: Cross‐Sectional Baseline Analysis From the MOBILIZE Trial

**DOI:** 10.1002/ejsc.70005

**Published:** 2025-07-09

**Authors:** Alessio Bricca, Grit Elster Legaard, Sofie Rath Mortensen, Jan Christian Brønd, Peter Gæde, Søren T. Skou

**Affiliations:** ^1^ Center for Muscle and Joint Health Department of Sports Science and Clinical Biomechanics University of Southern Denmark Odense Denmark; ^2^ The Research and Implementation Unit PROgrez Department of Physiotherapy and Occupational Therapy Næstved‐Slagelse‐Ringsted Hospitals Slagelse Denmark; ^3^ Centre for Physical Activity Research Rigshospitalet Copenhagen Denmark; ^4^ Research Unit for Exercise Epidemiology Centre of Research in Childhood Health Department of Sports Science and Clinical Biomechanics University of Southern Denmark Odense Denmark; ^5^ Department of Regional Health Research University of Southern Denmark Odense Denmark; ^6^ Department of Cardiology and Endocrinology Slagelse Hospital Slagelse Denmark

**Keywords:** chronic disease, exercise, immunology, lifestyle, metabolism

## Abstract

Physical activity (PA) has anti‐inflammatory effects, but its impact on individuals with multimorbidity (two or more chronic conditions) is unclear. We examined the association between device‐measured (i.e., accelerometers) PA and inflammatory biomarkers in people with multimorbidity. In a, preplanned, cross‐sectional analysis from the MOBILIZE trial, 214 participants with multimorbidity provided data on PA and inflammatory biomarkers including interleukin‐1 receptor antagonist (IL‐1ra), high‐sensitivity C‐reactive protein (hs‐CRP), tumor necrosis factor (TNF), and interleukin‐6 (IL‐6). The primary outcome was minutes per day of moderate‐to‐vigorous physical activity (MVPA). Adjusted robust regression models were used to evaluate associations, and BMI was explored as a mediator. Higher MVPA was associated with lower IL‐1ra levels, with a 2% reduction in IL‐1ra for each additional minute of MVPA per day. Participants with at least 10 min/day of MVPA had 33%–45% lower IL‐1ra levels compared to those with less than 1 min/day. Similar results were observed for secondary outcomes. BMI did not mediate the MVPA–IL‐1ra relationship. Even small increases in MVPA appear to be associated with low‐grade inflammation in individuals with multimorbidity. These findings support the promotion of PA in line with WHO guidelines for physical activity even in people with multimorbidity.

## Summary

- Physical activity (PA) has anti‐inflammatory effects, but its impact on individuals with multimorbidity (two or more chronic conditions) is unclear.
- In a, preplanned, cross‐sectional analysis from the MOBILIZE trial, we found that higher moderate‐to‐vigorous physical activity (MVPA) was associated with lower low‐grade systemic inflammation.
- Even small increases in MVPA may reduce low‐grade inflammation in individuals with multimorbidity.

## 1 Introduction

Multimorbidity, characterized by the presence of two or more chronic conditions, represents a major global health challenge, impacting about one‐third of adults (Skou et al. 2022). Those affected by multimorbidity face complex health issues, such as a higher likelihood of disability, diminished quality of life, and increased medical expenses (Dooley et al. 2023; Makovski et al. 2019; McPhail 2016; Nunes et al. 2016). Furthermore, multimorbidity is linked to heightened systemic inflammation, poor glycemic control, abnormal lipid levels, and elevated blood pressure, all of which contribute to the rapid advancement of chronic conditions and early death (Furman et al. 2019).

Physical activity has anti‐inflammatory effects (Furman et al. 2019; Pedersen and Saltin 2015; Gleeson et al. 2011), contributing to a reduced risk of morbidity (Furman et al. 2019; Pedersen and Saltin 2015; Gleeson et al. 2011; Shih et al. 2019). However, the majority of studies have predominantly focused on in vitro experiments, healthy individuals, or those with single chronic conditions (Furman et al. 2019; Pedersen and Saltin 2015; Gleeson et al. 2011). Only one cross‐sectional study has specifically examined individuals with multimorbidity (Poulsen et al. 2023). This study found that higher levels of physical activity, measured using accelerometers, were associated with lower levels of low‐grade systemic inflammation as indicated by high‐sensitive C‐reactive protein (hs‐CRP) (Poulsen et al. 2023). Nevertheless, these findings are limited by the fact that systemic low‐grade inflammation was assessed solely using hs‐CRP, and physical activity was measured only in terms of moderate‐to‐vigorous and light intensity. Additionally, BMI should be considered as a mediator in this relationship. Obesity contributes to inflammation through proinflammatory cytokines, whereas physical activity can reduce body fat and inflammation (Kawai et al. 2021; Khanna et al. 2022). Investigating BMI as a mediator can clarify whether the association between physical activity and systemic low‐grade inflammation is direct or primarily through reducing body fat. Therefore, further research is needed to confirm and expand these results by incorporating additional inflammatory biomarkers and a broader range of physical activity measures and analyses as well as BMI.

Such knowledge could be used to develop physical activity interventions to reduce risk of premature mortality and morbidity from multimorbidity (Makovski et al. 2019; McPhail 2016; Nunes et al. 2016; Fessler et al. 2023). Similarly, they may serve as the basis for investigating longitudinal associations between physical activity and low‐grade systemic inflammation. Therefore, we investigated if device‐based physical activity is associated with low‐grade biomarkers of inflammation in people with multimorbidity. Our primary hypothesis is that more time spent on moderate‐to‐vigorous physical activity (MVPA) is associated with lower IL‐1RA in people with multimorbidity. We chose MVPA as the primary exposure because the effect of physical activity on systemic low‐grade inflammation appears to be dose‐dependent with higher intensities promoting greater reductions (Legaard et al. 2023). We chose circulating IL‐1RA as the primary outcome because it appears to be more sensitive to physical activity than other common molecular biomarkers such as CRP and IL‐6 (Johansen et al. 2020).

## 2 Methods

Cross‐sectional study including patients from the MOBILIZE trial ClinicalTrials.gov registration: NCT04645732 (Skou et al. 2023), following a prespecified statistical analysis plan (https://osf.io/ypd4r).

Briefly, the MOBILIZE RCT investigates the effects of personalized exercise therapy and self‐management support in addition to usual care in patients with multimorbidity (Skou et al. 2023).

Two hundred twenty eight participants were randomized (1:1) to a 12‐week intervention, in addition to usual care or usual care alone (Skou et al. 2023). The program included 24 supervised exercise sessions (60 min/session) and 24 self‐management sessions (30 min/session) and is described elsewhere in detail (Bricca et al. 2022). Individuals diagnosed with at least two of the following conditions: Osteoarthritis of the knee/hip, hypertension, type‐2‐diabetes, depression, heart disease, and chronic obstructive pulmonary disease were recruited from six general practitioners, three psychiatric facilities, and seven hospital departments in the Region of Zealand, Denmark, through direct consultations, Facebook ads and local newspaper articles as well as other advertisement and posters/handouts. Details of the recruitment methods and eligibility criteria are published elsewhere (Skou et al. 2023). Additional participant characteristics, such as smoking habits and medication use, was self‐reported, age and sex were extracted from the participants social security number (CPR), and body mass index (BMI) was derived from height (cm) and weight (kg) during the baseline testing (Skou et al. 2023).

### 2.1 Device‐Based Physical Activity

The following variables were prespecified to capture the different measures of physical activity in this study.

- Minutes/day spent in moderate‐to‐vigorous physical activity (continuous and accelerometer‐based) (primary exposure)
- Steps per day (continuous and accelerometer‐based)
- Total physical activity minutes/day (continuous and accelerometer‐based)
- Minutes/day being physically active in walking, running, or biking (continuous and accelerometer‐based)

They were assessed using two Axivity AX3 (Axivity, Newcastle UK) accelerometers placed on the front of the right thigh and the wrist (nondominant). Participants were instructed to wear the accelerometers for seven consecutive days, including during sleep and water activities. Data were considered valid if participants provided at least 22 out of 24 h of wear time during a day for at least three weekdays and one weekend day (Herrmann et al. 2013; Petersen et al. 2022, 2020). The entire process of how data were processed is reported in a separate publication (Brønd et al. 2017). Briefly, physical activity intensities were generated using ActiGraph counts of 10 s for each intensity and step counts using a recently developed algorithm (Gustafsson et al. 2022). The thigh‐worn accelerometer has been used to determine activity behaviors (e.g., walking), whereas both accelerometers (tight and wrist) were used to determine intensities (Skotte et al. 2014). For adults aged 18 and over, the intensity cutoff points were: sedentary (< 100 counts), light (100 counts), moderate (3522 counts), and vigorous (6016 counts) (Brønd et al. 2019). MVPA was calculated as the sum of moderate and vigorous physical activity (Petersen et al. 2020). This method is highly valid for generating ActiGraph counts from raw acceleration data recorded with alternative devices (Brønd et al. 2017)

### 2.2 Molecular Biomarkers

The included biomarkers, that is, interleukin‐1 receptor antagonist (IL‐1ra pg/mL) (primary outcome), high‐sensitivity C‐reactive protein (hs‐CRP pg/mL), tumor necrosis factor (TNF pg/mL), and interleukin 6 (IL‐6 pg/mL), are considered the key variables to assess low‐grade systemic inflammation based on the literature (Furman et al. 2019; Calder et al. 2013). Additionally, given the complex etiology of low‐grade systemic inflammation, it is recommended to look at a set of biomarkers related to inflammation rather than single ones (Furman et al. 2019; Calder et al. 2013). The samples were stored at the local Departments of Clinical Biochemistry in Holbæk, Roskilde, Næstved, Slagelse, and Nykøbing Falster as well as at Rigshospitalet in Copenhagen. Fasting plasma samples were centrifuged and stored at −80°C until analyses. They were measured using immunoassay kits from Meso Scale Discovery, USA as follows: IL‐1Ra (V‐PLEX Cytokine Panel 2 (human) kit), CRP (V‐PLEX vascular injury Panel 2 (human) kit), TNF*α*, and IL‐6 (V‐PLEX Proinflammatory Panel 1 (human)). The participants were informed to fast by not eating or drinking anything except water for 10 h before the test.

### 2.3 Statistical Analysis and Covariates

We performed prespecified unadjusted and adjusted (for gender, smoking, body mass index (BMI), age, and number of chronic conditions) robust regression analyses, in STATA BE v18.0, and reported the results in beta coefficients (*β*) with 95% confidence intervals. *p* < 0.05 was considered statistically significant. This choice of the possible confounders was based on the literature for the association between physical activity and low‐grade systemic inflammation in in vitro, animal and human studies (Gleeson et al. 2011) and expert opinion (Supporting information S1: Figure S1). To improve the clinical interpretation of the findings, physical activity quartiles were used for assessing the association between MVPA and IL‐1ra. As prespecified in the statistical analysis plan, published before conducting the analyses, we explored the assumptions of the linear regression model, including normality of residuals, linearity, and homoscedasticity. Given that the assumptions of homoscedasticity and normality of the residuals were violated, despite using log‐transformed variables, (Figure S2 and Figure S3, respectively), we performed robust regression analysis to mitigate the impact of these assumption violations on regression estimates (Huang et al. 2012).

Additionally, to explore the role of BMI as a mediator on the primary outcome, we employed the Wald test, that is, the significance of the mediation effect of BMI on the association between physical activity and IL‐1 receptor antagonist (IL‐1ra). This test evaluates whether the indirect effect of the mediator (BMI) is significantly different from zero. The mediation effect of BMI was evaluated specifically between MVPA and IL‐1ra (primary outcome) to avoid performing multiple tests and improve the chance of spurious findings. Finally, we performed sensitivity analyses on the physical activity definitions to investigate the robustness of the main findings.

## 3 Results

Two hundred six out of two hundred twenty eight patients had valid molecular biomarkers and physical activity data and were included in this investigation. Reasons for the lack of valid physical activity data included suboptimal wear time due to the removal of accelerometers caused by skin reactions (itching or eczema) (*n* = 6), unknown reasons (*n* = 3), the device falling off and not being put back on (*n* = 1), and unwillingness to wear the device (*n* = 2). Additionally, one participant withdrew consent to participate in the study, two devices were not activated correctly, two experienced errors during data download, and three devices were lost in the mail. Participants had an average age of 70 years, with 44% female and 56% male participants. The mean BMI was 31, and the average number of chronic conditions per participant were seven. The most common chronic conditions were hypertension, type‐2 diabetes, and hip and knee osteoarthritis (Skou et al. 2024). Physical activity levels varied, with median MVPA ranging from 0.66 min/day in the lowest quartile to 30 min/day in the highest quartile. Biomarkers of low‐grade systemic inflammation showed median IL‐1ra levels at 201 pg/mL, hs‐CRP at 4.08 pg/mL, TNF at 1.22 pg/mL, and IL‐6 at 1.39 pg/mL (Table 1).

**TABLE 1:** Characteristic of the included patients.

Demographics	
Age (years)	70 (8)
Sex (% female/% male)	44/56
BMI	31 (6)
Number of conditions	7 (9) (range 2–19)
Smoking status (% yes/% no)	11/89
Physical activity variables	
MVPA/day^a^	5.4 (1.8–16.1) (*n* = 212)
MVPA/day^a^ (quartile 1, lowest)	0.66 (0.28–1.19) (*n* = 46)
MVPA/day^a^ (quartile 2)	3.4 (2.5–4.5) (*n* = 53)
MVPA/day^a^ (quartile 3)	10 (7–12) (*n* = 53)
MVPA/day^a^ (quartile 4, highest)	30 (21–41) (*n* = 53)
Steps per day^a^	4067 (2039–5967) (*n* = 212)
Total physical activity min/day	181 (129–236) (*n* = 212)
Minutes/day spent in light intensity physical activity	175 (167–184) (*n* = 212)
Minutes/day spent in moderate intensity physical activity	10 (8–12) (*n* = 212)
Minutes/day spent in vigorous intensity physical activity	1 (0–1) (*n* = 212)
Minutes/day spent in biking	0.96 (0.54–1.38) (*n* = 212)
Minutes/day spent in running	0.04 (0.01–0.09) (*n* = 212)
Minutes/day spent in walking	49 (45–53) (*n* = 212)
Minutes/day spent in biking, running, and walking^a^	46 (25–69) (*n* = 212)
Molecular biomarkers of low‐grade systemic inflammation	
IL‐1ra (pg/mL)^a^	201 (147–364) (*n* = 204)
hs‐CRP (mg/L)^a^	2.04 (1.091–4.86) (*n* = 206)
TNF (pg/mL)^a^	1.22 (0.98–1.67) (*n* = 205)
IL‐6 (pg/mL)^a^	1.39 (0.88–2.23) (*n* = 202)

^a^ Data are presented as mean (SD) or median (IQR), if not stated otherwise.

### 3.1 Association Between Physical Activity and Systemic Low‐Grade Inflammation

We found that higher MVPA was associated with lower concentration of IL‐1ra (primary outcome) (Table 2). This corresponds to a 2% lower level of IL‐1ra (pg/mL) per every additional MVPA minute/day. Similarly, participants in the third (MVPA min/day 10 (IQR 7–12)) and fourth (MVPA min/day 30 (IQR 21–41)) quartiles had IL‐1ra levels 33% and 45% lower, respectively, than those in the lowest MVPA quartile (MVPA min/day 0.66 (IQR 0.28–1.19) (Table 2). The sensitivity analyses investigating the relationship between minutes per day spent in light physical activity, moderate physical activity, vigorous physical activity, and walking were in line with the main analysis except for minutes per day spent in vigorous physical activity that was not associated with IL‐1Ra (Table S1).

**TABLE 2:** Association between physical activity and low‐grade inflammation (IL‐1ra, primary outcome) in people with multimorbidity.

IL‐1ra^a^ (unadjusted)				
Physical activity	Beta coeff.	95% CI	*p* value	Adj. *R* ^2^
MVPA min/day	−0.015	−0.023 to −0.006	< 0.001	0.10
Steps/day	−0.00008	−0.00012 to −0.00004	< 0.001	0.09
Total PA min/day	−0.003	−0.0052 to −0.0016	< 0.001	0.10
Min/day biking, running, and walking	−0.007	−0.011 to −0.003	< 0.001	0.09
MVPA/day^a^ (quartile 2)^b^	−0.07	−0.41 to 0.26	0.65	0.08
MVPA/day^a^ (quartile 3)^b^	−0.37	−0.66 to −0.08	0.01	
MVPA/day^a^ (quartile 4), highest)^b^	−0.54	−0.84 to −0.23	0.001	
**IL‐1ra** ^**a**^ **(adjusted)**				
MVPA min/day	−0.014	−0.022 to −0.006	0.001	0.30
Steps/day	−0.00007	−0.00011 to −0.00003	0.001	0.30
Total PA min/day	−0.003	−0.005 to −0.0009	0.005	0.29
Min/day biking, running, and walking	−0.007	−0.010 to −0.003	0.001	0.30
MVPA/day^a^ (quartile 2)^b^	−0.10	−0.41 to 0.21	0.53	0.29
MVPA/day^a^ (quartile 3)^b^	−0.39	−0.69 to −0.09	0.01	
MVPA/day^a^ (quartile 4, highest)^b^	−0.59	−0.96 to −0.23	0.001	

Abbreviation: Adj. = adjusted, Coeff = coefficient, IL‐1ra = interleukin‐1 receptor antagonist, MVPA = moderate‐to‐vigorous physical activity, and PA = physical activity.

^a^ Natural logarithmic transformation data were used for the analysis. The coefficients reflect the difference in the outcomes (log transformed) for each unit increase in the physical activity variable. Exponentiating the beta coefficient will provide the estimated percent difference in every molecular biomarker per physical activity variable. For example, a 1 min/day increase in MVPA is associated with an approximately 2% decrease in IL‐1ra (*e* ^−0.015^ ≈0.98).

^b^ The reference quartile is 1 (lowest MVPA/day).

Secondary analyses aligned with the primary findings (Table 3). Only MVPA's association with IL‐6 and hs‐CRP did not reach statistical significance in the adjusted analysis, despite the association being in the same direction as the primary analysis. BMI did not mediate the relationship between MVPA and IL‐1ra (*p* = 0.73) despite being associated with IL‐1ra (Table S2).

**TABLE 3:** Association between physical activity and low‐grade inflammation (secondary outcomes) in people with multimorbidity.

	Hs‐CRP^a^ (unadjusted)							TNF^a^ 1r^a^ (unadjusted)								IL‐6^a^(unadjusted)				
Physical activity	Beta coeff.		95% CI	*p* value		Adj.*R* ^2^		Beta coeff.			95% CI		*p* value	Adj. *R* ^2^		Beta coeff.		95% CI	*p* value	Adj. *R* ^2^
MVPA min/day	−0.010	−0.020 to −0.001			0.024		0.02		−0.008	−0.011 to −0.006		< 0.001			0.09		−0.004	−0.12 to 0.005	0.425	0.006
Steps/day	−0.00007	−0.0001 to −0.00002			0.007		0.04		−0.00006	−0.00007 to −0.00003		< 0.001			0.11		−0.00006	−0.0001 to −0.00002	0.004	0.06
Total PA min/day	−0.003	−0.0062 to −0.0008			0.010		0.05		−0.0015	−0.0024 to −0.0006		0.001			0.06		−0.003	−0.005 to −0.002	< 0.001	0.10
Min/day biking, running, and walking	−0.008	−0.013 to −0.002			0.006		0.05		−0.005	−0.006 to −0.003		< 0.001			0.10		−0.006	−0.009 to −0.003	0.001	0.07
Hs‐CRP^a^ (adjusted)									TNF^a^ 1r^a^ (adjusted)								IL‐6^a^ (adjusted)			
MVPA min/day	0.008	−0.018 to 0.022			0.124		0.07		−0.009	−0.012 to −0.006		< 0.001			0.14		−0.00004	−0.0090 to 0.0089	0.993	0.08
Steps/day	−0.00007	−0.00013 to −4.20e			0.037		0.08		−0.00005	−0.00007 to −0.00003		< 0.001			0.16		−0.00004	−0.00008 to −3.19e	0.035	0.11
Total PA min/day	−0.0028	−0.0057 to 0.00008			0.057		0.08		−0.001	−0.0024 to −0.0003		0.01			0.11		−0.003	−0.004 to −0.001	0.001	0.14
Min/day biking, running, and walking	−0.007	−0.013 to −0.0008			0.028		0.08		−0.005	−0.007 to −0.002		< 0.001			0.15		−0.005	−0.008 to −0.001	0.009	0.12

Abbreviation: Adj. = adjusted, Coeff = coefficient, hs‐CRP = high‐sensitivity C‐reactive protein, IL‐6 = interleukin 6, MVPA = moderate‐to‐vigorous physical activity, PA = physical activity, and TNFα1ra = tumor necrosis factor α receptor antagonist.

^a^ Natural logarithmic transformation data were used for the analysis. The coefficients reflect the difference in the outcomes (log transformed) for each unit increase in the physical activity variable. Exponentiating the beta coefficient will provide the estimated percent difference in every molecular biomarker per physical activity variable.

## 4 Discussion

This is the first study, to the best of our knowledge, to investigate the association between device‐based physical activity and a comprehensive set of low‐grade systemic inflammation biomarkers in people with multimorbidity. The result of this cross‐sectional study can serve as a foundation for investigating the causal–effect relationship between physical activity and systemic low‐grade inflammation in randomized controlled trials including people with multimorbidity.

We found that every minute of additional MVPA is associated with lower low‐grade systemic inflammation. However, at least 10 min/day of MVPA were linked to lower inflammation levels compared to those with less than 1 min/day of MVPA. Yet, the lowest inflammation level was observed in the group performing 30 min/day of MVPA. This is in line with the optimal physical activity level recommended by the WHO guidelines for health benefits (Bull et al. 2020), and randomized controlled trials in people with diabetes suggesting that higher physical activity is associated with lower levels of low‐grade inflammation (Johansen et al. 2020). In contrast, our study differs from previous cross‐sectional studies (Booker et al. 2022; Chastin et al. 2015) on physical activity and systemic low‐grade inflammation by demonstrating that even lower intensities than moderate‐to‐vigorous physical activity (MVPA) may contribute to reducing inflammation. However, direct comparisons of these findings should be interpreted with caution due to differences in the populations studied and the specific aims of the studies.

Our findings also suggest that other forms of physical activity than MVPA, including steps per day, low‐intensity activities, and specific activities, such as walking, are significantly associated with lower levels of IL‐1ra, an anti‐inflammatory marker. By contrast, minutes per day spent in vigorous physical activity was not associated with IL‐1ra due to the wide 95% CI of association coefficients. The lack of mediation by BMI in this relationship might be due to BMI being a general measure that does not capture fat distribution or muscle mass (Okorodudu et al. 2010), which are more directly related to inflammation (Kivimäki et al. 2017). Furthermore, the lack of association between MVPA with IL‐6 and hs‐CRP in the adjusted analysis, despite the association being in the same direction as the primary analysis, may be due to the small sample size or lower sensitivity of these biomarkers to physical activity. This can allow clinicians to encourage patients to engage in any type of physical activity, tailoring to their abilities and preferences, to achieve these anti‐inflammatory benefits.

Reflecting on the mechanisms, this might be attributed to a decrease in visceral fat mass, leading to a reduced release of adipokines from adipose tissue, and/or the creation of an anti‐inflammatory environment with each physical activity bout (Gleeson et al. 2011). As an example, physical activity may reduce low‐grade systemic inflammation through several mechanisms, including the release of interleukin‐6 (IL‐6) from muscles, which boosts levels of IL‐1 receptor antagonist (IL‐ra) (Steensberg et al. 2003). However, given the cross‐sectional nature of our study and focus, we were not able to assess such mechanisms (Gleeson et al. 2011; Shih et al. 2019).

Limitations of our study include: (i) inability to adjust for medication use (which were reported inconsistently) or other factors, such as diet and alcohol consumption, which may influence inflammation; (ii) small sample size, though a power calculation suggests that a 214‐participant sample could detect a 6% increase in *R* ^2^ with 90% power and a 5% alpha; and (iii) the cross‐sectional design of this investigation prevents causality inference. Beyond these, other limitations include potential confounders, such as diet, socioeconomic status, and genetic predispositions, and the lack of longitudinal data to understand the sustained effects of physical activity on inflammation and overall health in individuals with multimorbidity.

## 5 Conclusion

Physical activity appears to be associated with systemic low‐grade inflammation also in people with multimorbidity. Overall, this knowledge supports the development of lifestyle interventions targeting low‐grade inflammation in people with multimorbidity as well as clinicians to prescribe physical activity in line with the WHO guidelines for physical activity and sedentary behavior, which may ultimately reduce the risk of early death in this population (Furman et al. 2019; Gleeson et al. 2011; Shih et al. 2019). Additionally, these results serve as the basis for investigating whether intervention aimed at increasing physical activity may have anti‐inflammatory effects in people with multimorbidity.

## Ethics Statement

The RCT was approved by the Regional Committee on Health Research Ethics for Region Zealand (SJ‐857) on May 28, 2020 and is conducted in agreement with the Helsinki Declaration. Informed consent material is available in Danish with the approved protocol. If a participant sustains any trial‐related harm, he or she will be covered by the Danish law. Furthermore, the RCT was approved by the Danish Data Protection Agency of the Region of Zealand (REG‐015‐2020) and was preregistered at ClinicalTrials.gov (NCT04645732).

## Consent

The authors have nothing to report.

## Conflicts of Interest

STS is an associate editor of the Journal of Orthopedic and Sports Physical Therapy and has received personal fees from Munksgaard, TrustMe‐Ed, and Nestlé Health Science, all of which are outside the submitted work. He is a cofounder of Good Life with Osteoarthritis in Denmark (GLA:D), a not‐for profit initiative hosted at University of Southern Denmark aiming at implementing clinical guidelines for persons with osteoarthritis in clinical practice. The authors declare that there are no other conflicts of interest. AB has received personal fees from TrustMe‐Ed and PhisioVit srl, all of which are outside the submitted work.

## Supporting information

Supporting Information S1

Figure S2

Figure S3

Table S1

Table S2

## Data Availability

Data will be made available upon reasonable request.
