# Supplementary material for: Device‐Based Physical Activity and Low‐Grade Inflammation in People With Multimorbidity: Cross‐Sectional Baseline Analysis From the MOBILIZE Trial
Source: Eur J Sport Sci. 2025 Jul 9;25(7):e70005. doi: 10.1002/ejsc.70005 (PMC12239932; doi:10.1002/ejsc.70005)
Supplement: Supplementary file 1 — Supporting Information S1 [file EJSC-25-e70005-s002.docx]

Based on evidence for the association of physical activity and low-grade inflammation and expert opinion (1) we have co-developed the following directed acyclic graph (DAG). DAG is an approach for identifying confounding variables that require conditioning when estimating causal effects.


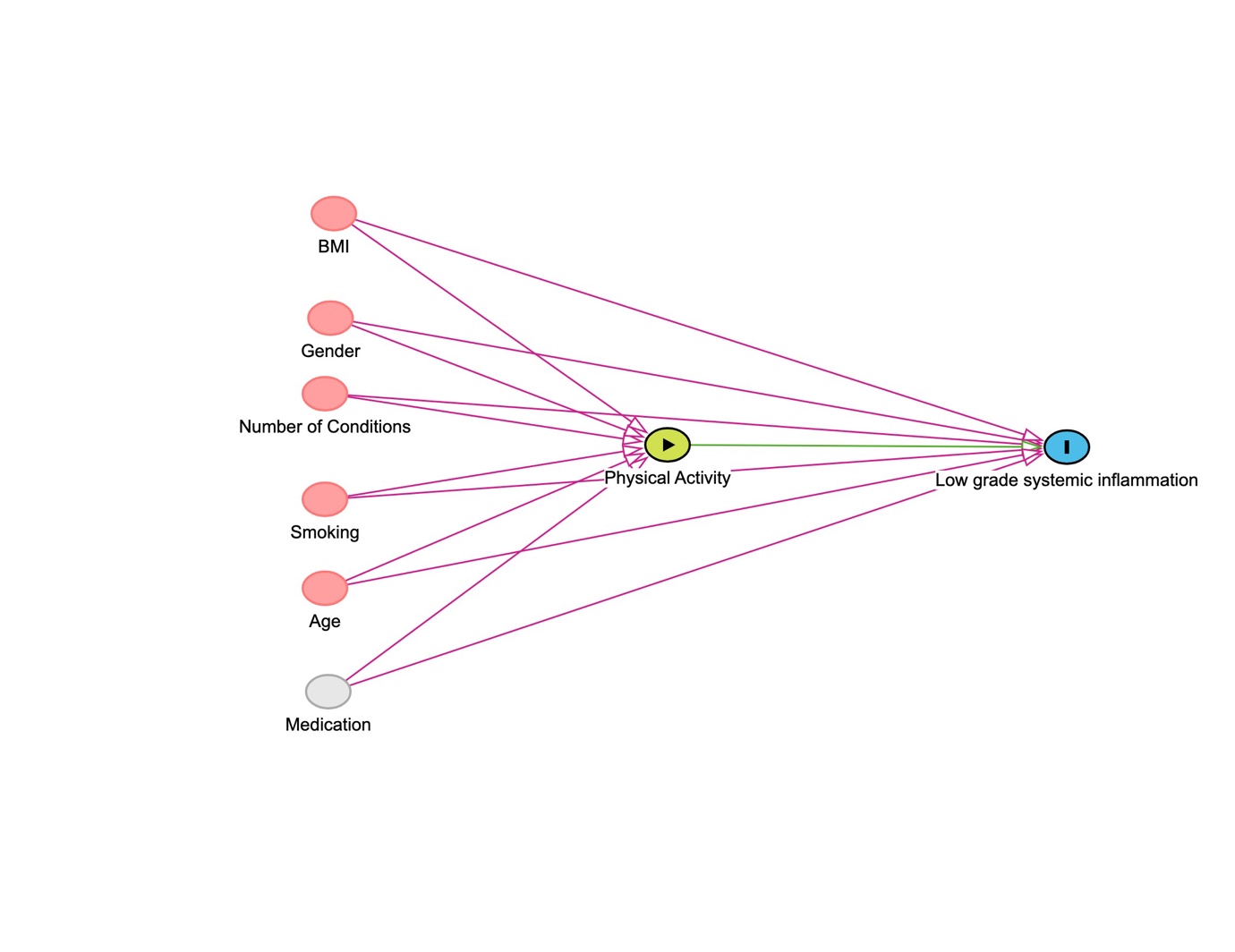


**Supplementary Figure 1**. The assumed DAG of potential factors associated with physical activity and low-grade systemic inflammation. Outcome (blue circle) = Low grade systemic inflammation. Exposure (yellow circle) = Physical activity. Unobserved potential confounder (grey circle) = = Medication (we anticipated to include medication as a potential confounder and stratifying it into glucose-lowering, blood pressure-lowering, cholesterol-lowering, and anti-inflammatory. However, given the lack of consistency in self-reporting by the included participants we were unable to include it in the model). Potential confounders (red circles). Green arrows indicate a casual path. Red arrows indicate a biasing path.

**Reference**

1. Gleeson, M., Bishop, N., Stensel, D. et al. The anti-inflammatory effects of exercise: mechanisms and implications for the prevention and treatment of disease. Nat Rev Immunol 11, 607–615 (2011). https://doi.org/10.1038/nri3041
