# Supplementary material for: Device‐Based Physical Activity and Low‐Grade Inflammation in People With Multimorbidity: Cross‐Sectional Baseline Analysis From the MOBILIZE Trial
Source: Eur J Sport Sci. 2025 Jul 9;25(7):e70005. doi: 10.1002/ejsc.70005 (PMC12239932; doi:10.1002/ejsc.70005)
Supplement: Supplementary file 2 — Figure S2 [file EJSC-25-e70005-s003.docx]

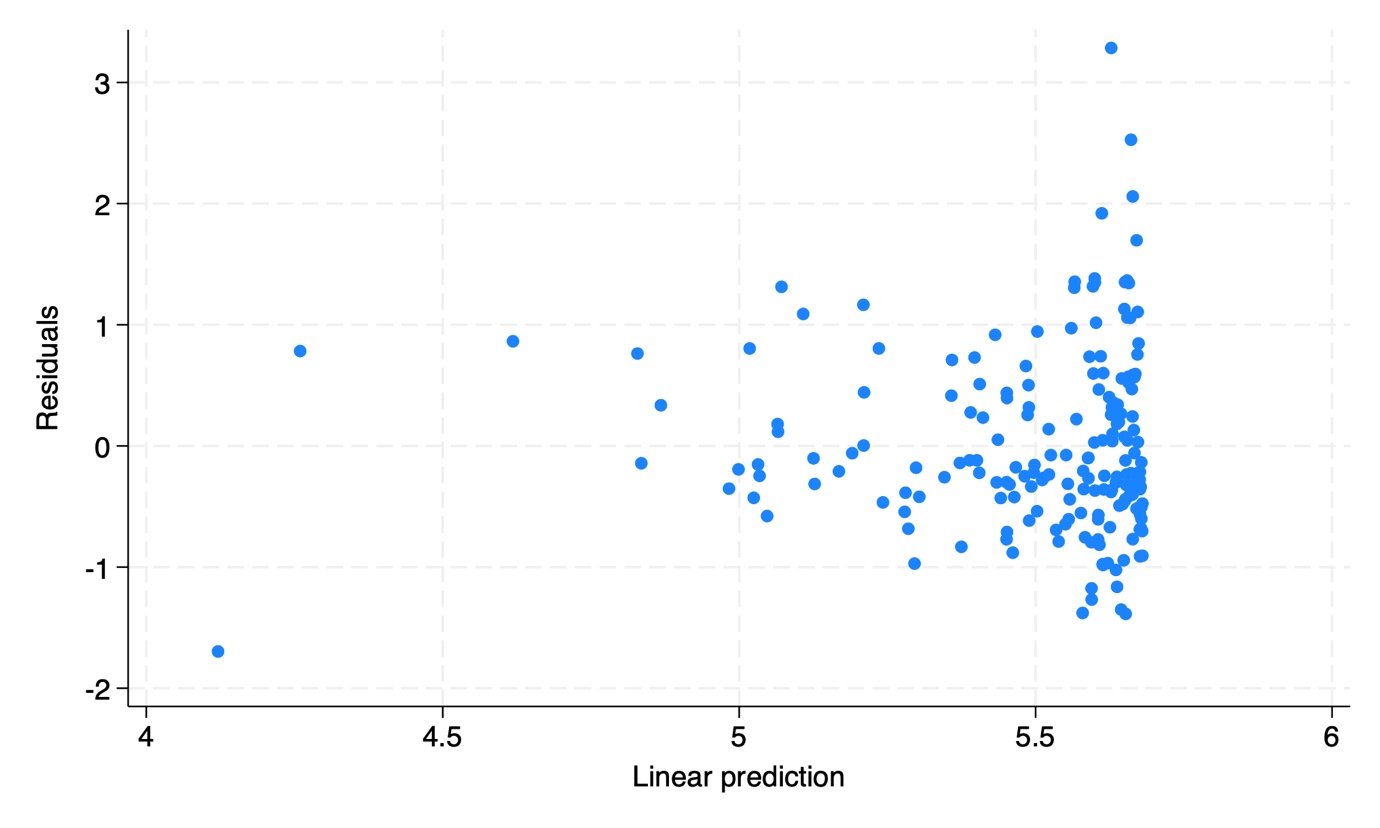


**Supplementary Figure 2. Homoscedasticity.** This plot displays the residuals (errors) from the linear regression model on the y-axis against the fitted values (predicted values) on the x-axis. Each point represents an observation from the dataset. The assumption of homoscedasticity in linear regression requires that the residuals have constant variance across all levels of the independent variable(s). When this assumption is violated, despite using a log-transformed variable, as indicated by the figure above, it means that the residuals' variance changes with the fitted values and one of the reasons we used robust regression analyses instead.
