# Supplementary material for: Device‐Based Physical Activity and Low‐Grade Inflammation in People With Multimorbidity: Cross‐Sectional Baseline Analysis From the MOBILIZE Trial
Source: Eur J Sport Sci. 2025 Jul 9;25(7):e70005. doi: 10.1002/ejsc.70005 (PMC12239932; doi:10.1002/ejsc.70005)
Supplement: Supplementary file 3 — Figure S3 [file EJSC-25-e70005-s004.docx]

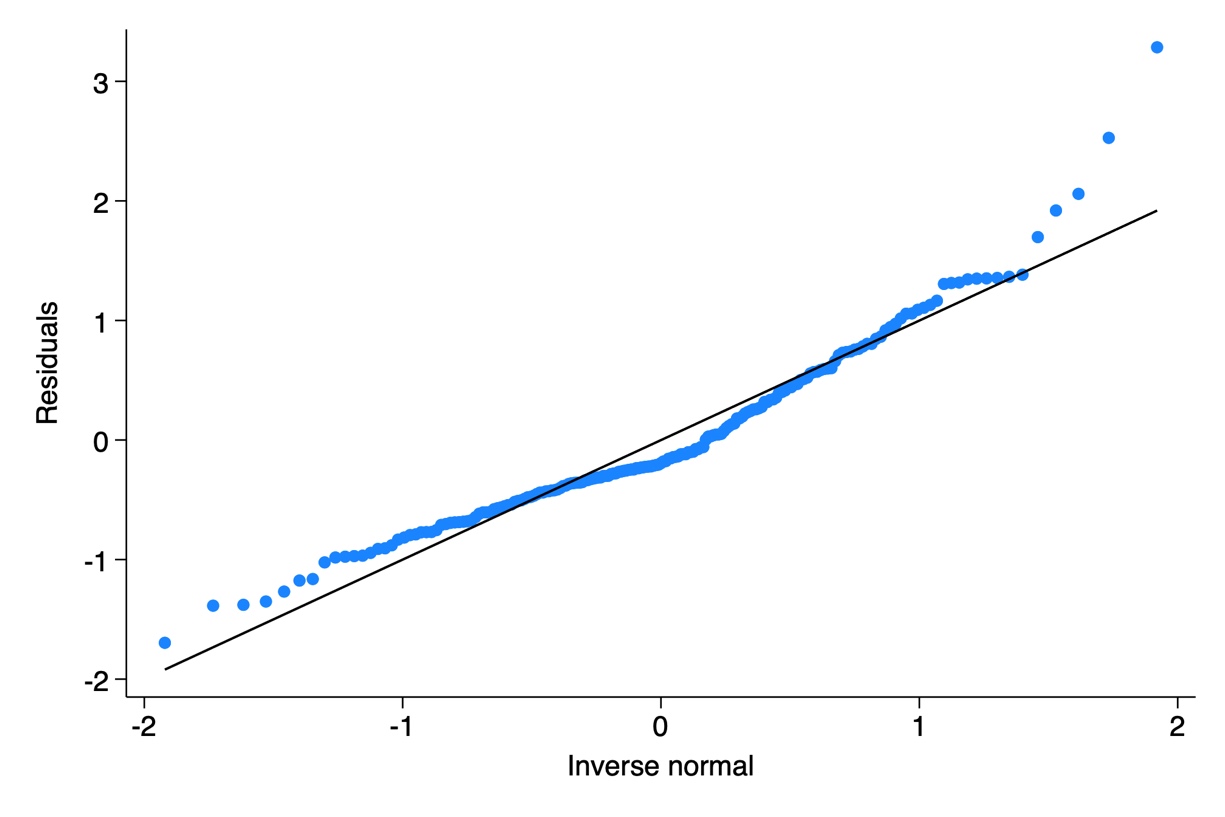


**Supplementary Figure 3. Normality of residuals for linear regression.** The plot includes a histogram of the residuals, a Q-Q plot comparing the residuals to a normal distribution, and the p-value from the Shapiro-Wilk test. The Shapiro-Wilk test yielded a p-value of (p = 0.00000), indicating that the residuals significantly deviate from a normal distribution.
