# Supplementary material for: Device‐Based Physical Activity and Low‐Grade Inflammation in People With Multimorbidity: Cross‐Sectional Baseline Analysis From the MOBILIZE Trial
Source: Eur J Sport Sci. 2025 Jul 9;25(7):e70005. doi: 10.1002/ejsc.70005 (PMC12239932; doi:10.1002/ejsc.70005)
Supplement: Supplementary file 4 — Table S1 [file EJSC-25-e70005-s005.docx]

**Supplementary Table 1. Sensitivity analyses on the physical activity classification.**

|  | **IL-1ra^a^ (unadjusted)** | | | | |
| --- | --- | --- | --- | --- | --- |
|  | **Beta Coeff.** | **95% CI** | ***P* value** | **Adj. R^2^** |  |
| **Light physical activity (min/day)** | -.00007 | -.0001 to -.00004 | <0.001 | 0.11 |  |
| **Moderate physical activity (min/day)** | -.0003 | -.0004355 to -.0001 | <0.001 | 0.10 |  |
| **Vigorous physical activity (min/day)** | -.0002 | -.0007 to .0002 | 0.36 | 0.01 |  |
| **Walking (min/day)** | -.0001 | -.0002 to -.00006 | <0.001 | 0.09 |  |
|  | **IL-1ra ^a^ (adjusted)** | | | | |
|  | **Beta Coeff.** | **95% CI** | ***P* value** | **Adj. R^2^** |  |
| **Light physical activity (min/day)** | -.00007 | -.0001 to -.00004 | <0.001 | 0.30 |  |
| **Moderate physical activity (min/day)** | -.0003 | -.0004 to -.0001 | <0.001 | 0.32 |  |
| **Vigorous physical activity (min/day)** | -.00004 | -.0005 to .0005 | 0.87 | 0.24 |  |
| **Walking (min/day)** | -.0001 | -.0002 to -.00005 | 0.001 | 0.30 |  |

^a^Natural logarithmic transformation data was used for the analysis. The coefficients reflect the difference in the outcomes (log transformed) for each unit increase in the physical activity variable. Exponentiating the beta coefficient will provide the estimated percent difference in every molecular biomarker per physical activity variable.
