# Supplementary material for: Device‐Based Physical Activity and Low‐Grade Inflammation in People With Multimorbidity: Cross‐Sectional Baseline Analysis From the MOBILIZE Trial
Source: Eur J Sport Sci. 2025 Jul 9;25(7):e70005. doi: 10.1002/ejsc.70005 (PMC12239932; doi:10.1002/ejsc.70005)
Supplement: Supplementary file 5 — Table S2 [file EJSC-25-e70005-s001.docx]

**Supplementary Table 2**. Association between BMI and IL-1ra.

|  | **IL-1ra^a^ (unadjusted)** | | | | |
| --- | --- | --- | --- | --- | --- |
|  | **Beta Coeff.** | **95% CI** | ***P* value** | **Adj. R^2^** |  |
| **BMI** | 0.06 | 0.04 to 0.08 | <0.001 | 0.23 |  |
|  | **IL-1ra ^a^ (adjusted)** | | | | |
|  | **Beta Coeff.** | **95% CI** | ***P* value** | **Adj. R^2^** |  |
| **BMI** | 0.05 | 0.03 to 0.07 | <0.001 | 0.30 |  |

^a^Natural logarithmic transformation data was used for the analysis. The coefficients reflect the difference in the outcomes (log transformed) for each unit increase in the physical activity variable. Exponentiating the beta coefficient will provide the estimated percent difference in every molecular biomarker per physical activity variable. BMI=body mass index.
